# Weather Conditions and the Risk of Tomato Spotted Wilt Virus (TSWV) in Tomato Producing Areas in Southern Ghana

**DOI:** 10.1002/pei3.70121

**Published:** 2026-02-16

**Authors:** Rebecca Sarku, Maxwell Amartey Adjaottor, Etse Lossou

**Affiliations:** ^1^ University of Leeds Leeds UK; ^2^ Ministry of Food and Agriculture Accra Ghana; ^3^ Cooperative Rabobank U.A. Utrecht the Netherlands

**Keywords:** early spring weather factors, Ghana, smallholder farming, spotted wilt, tomato farming

## Abstract

Tomato farmers in southern Ghana incur losses due to the infection of crops by the tomato spotted wilt virus (*Orthotospovirus tomatomaculae*). The occurrence of the virus varies among individual tomato crops, influenced by the vector population and changing weather patterns. This study investigates the effect of early spring weather conditions (February–March) on the risk of occurrence of tomato spotted wilt virus in tomato‐producing regions in southern Ghana. Interviews and focus group discussions were conducted with farmers in the Ada East and West Districts to generate information on the period, frequency and the severity of tomato spotted wilt virus occurrences for the period 2020–2023. Meteorological data for the focal years were analyzed to identify the weather variables that influenced the occurrence of the virus, including the specific months of occurrence. Logistic regression analysis shows a high correlation between temperature and the occurrence of TSWV. Findings indicate that high temperature (29°C–32°C) recorded between January and March in the area correlate with farmers' observations of the virus. Observation by farmers that the viral infection started from March imply that it could have originated in the nursery and transported to the farm. The practical implication of the findings for management requires that farmers plant resistant varieties, frequent scouting for thrips, adoption of hygienic cultural practices, while agricultural extension agents and meteorological stakeholders provide farmers with timely agronomic information and accurate weather forecasts to enable early detection and response.

## Abstract 2

This study aims to investigate the effect of early spring weather conditions (February–March) on the risk of occurrence of spotted wilt in tomato‐producing regions in southern Ghana.

## 1 Introduction

Tomato ( *Solanum lycopersicum* ) is an important food crop with varied applications, including food preparation. Tomatoes hold greater significance among vegetables in Ghana compared to other crops, as they are consumed daily by every household (Schippers 2000; Lamptey and Koomson 2021; Nkansah et al. 2021). With a relatively short maturity period and an extended production cycle, often spanning up to 1 year, this crop is economically viable for numerous farmers (Vijitha and Mahendran 2012). Tomatoes flourish across various cropping systems and climatic conditions, ranging from temperate to warm and humid tropical environments (Shafiwu et al. 2022).

In Ghana, tomato production is predominantly carried out on a small scale and follows a seasonal pattern, with few large‐scale cultivation projects under irrigation (Opoku et al. 2021; Awu et al. 2023). The cultivation of the crop yields an annual production of 366,772,000 tons during the peak harvest season in Ghana (Owureku‐Asare et al. 2022). However, the average tomato yield is significantly below the potential yield of 20 tons per hectare (Ministry of Food and Agriculture (MOFA) 2017). Moreover, about 440,000 tons of tomatoes are consumed annually. While domestic production has been robust, it has not fully met the demand, which stood at approximately 420,000 tons (FAO 2019). The production shortfall necessitates annual imports of around 100,000 tons to satisfy local demand (FAO 2019). This situation has contributed to the annual import of tomatoes from neighboring countries like Burkina Faso, Mali, and Togo, resulting in a strain on the economy (Okali and Sumberg 2012; Venus et al. 2013; Balanaa et al. 2019).

The substantial yield gap observed in Ghana's tomato cultivation stems from various constraints, encompassing biotic and abiotic factors. Abiotic factors include unpredictable rainfall, high temperatures, and subpar soil conditions (Kugblenu et al. 2013; Osei‐Bonsu et al. 2022). Tomatoes grown in open fields face exposure to adverse weather conditions, with heightened risks of insect infestations and pest attacks. Despite efforts to increase cultivation, factors such as pests, diseases, and climate variability contribute to reductions in crop yield (Melomey et al. 2022).

A disease condition such as tomato spotted wilt virus (TSWV) (*Orthotospovirus tomatomaculae*) is a significant biotic factor severely affecting tomato production globally. TSWV is considered a global pest because it occurs across temperate, tropical, and subtropical regions, impacting a wide variety of crops and causing substantial economic losses worldwide. It also has the broadest natural and artificial host range among plant viruses, infecting more than 1000 confirmed species. Due to its extensive impact and scientific significance, TSWV ranks among the top 10 most important plant viruses globally (Parrella et al. 2003; Scholthof et al. 2011). It infects tomato leaves, stems, and roots at any stage of development and potentially destroys the entire tomato field within a few days. Common foliar symptoms include stunting, chlorotic spots, concentric rings, necrotic spots, and yellowing (Almási et al. 2020). The western flower thrips ( *Frankliniella occidentalis* —*Pergande*) and tobacco thrips ( *Frankliniella fusca* —*Hinds*) are the major vectors associated with transmitting TSWV in tomato fields (Broughton et al. 2004; Bielza 2008). The virus is spread by the thrip vector, which develops and spreads rapidly under high relative humidity, moderate temperature, and substantial rainfall. The impact of thrip infestation on a crop depends on the suitability of weather conditions for population growth, among many other factors (Broughton et al. 2004).

The link between rainfall and temperature and thrips and spotted wilt development has been documented in several studies (Waiganjo et al. 2008; Morsello et al. 2008; Olatinwo et al. 2009; Olatinwo et al. 2012; Riley et al. 2012; Chappell et al. 2013; Olatinwo and Hoogenboom 2014). Heavy rainfall was reported to have a negative effect on the survival of thrips larvae (Ibrahim and Adesiyun 2010) and the flight of adult thrips (Barbosa et al. 2019), whereas increased temperatures during spring were associated with increased thrips activity and population growth (Pearsall and Myers 2001; Morsello et al. 2010; Aliakbarpour et al. 2010; Mila 2011). Also, low temperatures and rains are detrimental to thrips colonization (Miray and Mehmet 2012; Karuppaiah et al. 2023). Thrips often migrate to cropping fields during spring after overwintering on uncultivated plants or alternative hosts (Swain 2019; He et al. 2020; Sampson et al. 2021). The timing of the preparation of seeds in the nursery and transplanting in relation to the emergence and movement of thrips to the farm can significantly contribute to the incidence of TSWV during the cropping season (Culbreath et al. 2003).

Stormy weather conditions have been linked to the mass flights of thrips. Weather fronts and incipient thunderstorms have been reported to discourage the mass flight of thrips, thereby resulting in high densities above the soil surface due to the thrips' landing attempts (Kirk 2004; Tashauna 2008). Another study found that the number of thrips captured in flight positively correlates with the number of wet days or days with precipitation (Morsello et al. 2008; Waiganjo et al. 2008). The population of the adult thrip vectors (*F. occidentalis and F. fusca*) was reported to be greater for early planting in April or late planting in June compared to planting in May (Kirk 2004; LaTora et al. 2022). At the same time, there were higher levels of spotted wilt associated with early‐ and late‐planted peanut compared to those planted during the middle of the season (Brown et al. 2005; Olatinwo et al. 2008; Nuti et al. 2014).

Farmers apply various strategies, such as deploying resistant varieties, fungicides, and cultural practices like early planting and mechanical haulm killing in managing TSWV, including repeated agrochemical applications (Blaeser et al. 2004; Bielza 2008; Batuman et al. 2020). Yet, management practices such as agrochemical applications currently adopted by farmers are ineffective. Once a plant is infected, there is no cure, highlighting the importance of vector growth limitation as a main management practice. With the rapid expansion of tomato production in Ghana, and the prevalence of TSWV, controlling the spread of the virus is paramount to enhance the tomato production (Lamptey et al. 2013; Asare‐Bediako, Wonkyi, et al. 2017; Asare‐Bediako, Mensah‐Wonkyi, et al. 2017; Obeng et al. 2018; Agbaglo et al. 2020; Opoku et al. 2021; Osei‐Bonsu et al. 2022; Awu et al. 2023). Elsewhere, practices for managing spotted wilt rely on using a preseason TSWV risk index. The risk index assesses the spotted wilt risk levels associated with production practices to help growers avoid high‐risk situations (Brown et al. 2005). The index includes cultivar, planting date, plant population, type of insecticide application, row pattern, tillage system, and presence or absence of classic herbicide (Olatinwo et al. 2009; Barbosa et al. 2019). However, the current formulation of the index does not include a weather component, nor does it account for early spring (i.e., February to March) weather parameters for predicting the prevalence of thrips and the expected level of spotted wilt intensity.

The challenge of pest and disease infestation of tomato production has prompted research in the sector in Ghana. Yet, a focus on the TSWV is missing. Attempts that aimed at researching tomato pathogen conditions focused broadly on the occurrence of diseases like tomato yellow leaf curl virus, tomato mosaic virus, bacterial wilt, bacterial spot, and early blight (Lamptey et al. 2013; Asare‐Bediako, Wonkyi, et al. 2017; Asare‐Bediako, Mensah‐Wonkyi, et al. 2017; Obeng et al. 2018; Agbaglo et al. 2020; Opoku et al. 2021; Osei‐Bonsu et al. 2022; Awu et al. 2023). Moreover, most research on tomato production in Ghana have been focused on the forest and Guinea Savanna agroecological zones (see, for instance, Dorward et al. 2003; Zseleczky et al. 2014; Lamptey and Koomson 2021; Nkansah et al. 2021; Benabderrazik et al. 2022; Amankwaa‐Yeboah et al. 2023). Tomato is also produced in commercial quantities in the Coastal Savanna region of Ghana. This study is therefore focused on the Ada Districts, to assess the effect of early spring weather conditions on the risk of TSWV in tomato producing areas in southern Ghana. In this study early spring refers to the period from February to March. Specifically, the study aims to identify weather variables for predicting spotted wilt occurrence in tomato fields. We propose that early spring weather parameters affect the risk of TSWV occurrence, presumably due to the impact of weather conditions on thrips vector (viruliferous) activities and development.

This paper is organized as follows: Section 2 presents a literature review on tomato production in Ghana. In Section 3, we present the context of the research including the research method. In Section 4, the results are presented, and discussions in Section 5, followed by a concluding section.

## 2 Empirical Literature on Pest and Disease Management in the Tomato Production Sector in Ghana

Tomato production spans various agroecological zones in Ghana, with regions like Tono, Vea, and Techiman recognized for large‐scale cultivation (Adu‐Dapaah and Oppong‐Konadu 2002). Seasonal producing regions like Navrongo and the Techiman areas shape the supply chain dynamics, with Techiman serving as a major source during the rainy season and imports filling transitional periods (Amikuzuno and von Cramon‐Taubadel 2012; Zseleczky et al. 2014). Tomato production involves small family farms predominantly using rain‐fed cultivation, heavily reliant on inputs like fertilizers and pesticides.

Agro‐climatic conditions vary between regions like Navrongo and Techiman, impacting production. Tomatoes' susceptibility to extreme climatic events poses challenges for stability and productivity, with the sector experiencing pronounced seasonality and price volatility (Guodaar et al. 2016; Amikuzuno and von Cramon‐Taubadel 2012). Climate variability poses a significant threat to tomato production, impacting yields and prompting importation to meet local demand (Guodaar et al. 2017). Ghana lags in yield compared to countries like Burkina Faso, attributed to multiple constraints including diseases, pests, and limited access to inputs (Melomey et al. 2022; Awu et al. 2023). In a related study, Benabderrazik et al. (2022) investigated how Ghanaian tomato farmers navigate double exposure from climate and market‐related shocks, stressing the urgency for comprehensive strategies to enhance resilience.

The environmental challenges affecting tomato production in Ghana are multifaceted, with empirical studies highlighting various factors contributing to suboptimal yields. Asare‐Bediako et al. (2007) noted that high humidity and abundant rainfall in Ghana create favorable conditions for pests and diseases, acting as impediments to successful tomato cultivation. Lamptey et al. (2013) investigated viral diseases' prevalence in major tomato‐growing areas. Other studies evaluated tomato varieties' resistance to tomato yellow leaf curl virus (TYLCV) (Segbefia et al. 2018); and the interactions between fungi and nematodes affecting tomato crops (Agbaglo et al. 2020; Opoku et al. 2021). Empirical research on tomato farming in Ghana has provided valuable insights into pest and disease occurrence; yet, there is a knowledge gap on the TSWV, thereby justifying its selection to examine the relationship between its spread and environmental conditions.

### 2.1 Study District

The data for the study was collected from farming households in the Ada districts of the Greater Accra Region of Ghana, a hub for tomato production near Accra, Ghana. Situated at an altitude of 4.45 m (14.6 ft) above sea level, the Ada area experiences a tropical wet and dry or savanna climate. The district's average annual temperature is 27°C (82.18°F). Ada typically receives approximately 750–800 mm of rainfall annually. In the Ada Districts, incidences of TSWV infections were reported in tomato and pepper fields in 2017. Significant economic losses have been reported by tomato farmers caused by TSWV in the Ada districts. The districts were purposively selected because they are predominantly tomato producing, rural, and agrarian districts, where tomatoes and other vegetables like pepper, onion, carrot, and traditional staple crops are supplied to the urban markets in the Greater Accra Region in Ghana. The Ada East District is subdivided into three zones: Big‐Ada, Kasseh, and Ada‐Foah, while Ada West is made up of two council areas (Sege and Anyamam). In the Ada East District, we selected Toje, Kpodokope, Manaikpo, Dogo, Anyarkpor, and Angorsekope. In the Ada West District, the selected communities were Sege, Hwakpo, Kuloedor, Segese, Addokope, and Toflokpo.

### 2.2 Data Collection

In total, 73 farmers were interviewed in the Ada Districts from January–March 2024—and at least 4 farmers from each community to identify the period TSWV occurrence, month/year of occurrence of TSWV including farmers' observations on the severity of the virus for specific seasons. The farmers were selected based on their availability, cultivation of tomatoes, and knowledge about TSWV. We also identified the characteristics of tomato farmers as follows: rainfed farmers, both rainfed and irrigation farmers, and only irrigation. There were some groups of farmers who cultivate tomato from spring (March/April) until the end of autumn (October–November). Farmers are knowledgeable and able to recount incidents based on experiences related to climatic conditions and farm practices. However, there is the likelihood to forget certain events (e.g., severity of the occurrence of the pathogen) (Singh 2014); hence, we decided to limit the analysis to the immediate past 4 years (2020–2023) (see Appendix S1). The period selected coincided with reports of the emergence of thrips and TSWV in the area. Additionally, tomato farmers were selected to generate knowledge about TSWV through focus group discussions organized for both male and female farmers. A total of eight farmers from different communities in the Ada East District were brought together at Agorsekope, while another focus group in the Ada West District was organized at Nakomkope. The participants were farmers with extensive experience in tomato production. In total, four focus group discussions were held across the Ada districts.

### 2.3 Ethical Considerations and Approval

This study was approved by the Ethics Committee of the University for Development Studies, Tamale (UDSRB/244/24), and all respondents provided verbal informed consent before the interviews were conducted.

### 2.4 Planting and Harvesting Dates of Tomatoes

Farmers adopt a decision strategy for sowing seeds and transplanting seedlings stages, described as early planters (March/April), mid planters (May), transplanting in batches (bi‐weekly), and late planters (August). Hence, interviews indicate planting dates ranged from: (i) prior to 1 April; (ii) 1–15 April; (iii) 16 April–30 May; (iv) 1–30 June; (v) 1 June–31 July; and (vi) 1 August–30 September. Current practices in irrigation areas in the Ada East District typically commence with land clearing in February and conclude with tomato harvesting in July. However, rainfed farmers slightly lag in transplanting seedlings due to dependence on rainfall for farming. Consequently, seedling transplanting begins in April–May and harvesting spans from late July to mid‐September in the districts. Also, for early transplanting of seedlings in late summer (August/September). Farmers opine that early flowering, when temperatures are high, results in high flower abortion rates and fruit drop, leading to decreased yields. The growth of the tomato crop is also adversely affected by dry spells (drought) which usually occur in June/July during the main farming season (i.e., April—July).

### 2.5 Data Preparation

Following the transcription of the data collected from the field into excel, Python was used to clean the data. This involved removing any inconsistencies, handling missing values, and standardizing the data to ensure uniformity. The preprocessing steps were crucial in preparing the dataset for subsequent analysis, ensuring reliability and accuracy of the results. In addition to the field data collection, this study also relied on historical rainfall and temperature data spanning from 1960 to 2023. The historical data was extracted from ERA 5 Copernicus Climate Change Service (C3S) (2017), a state‐of‐the‐art reanalysis dataset produced by the European Centre for Medium‐Range Weather Forecasts (ECMWF). ERA 5 provides hourly estimates of several atmospheric, land, and oceanic climate variables. It is widely recognized for its high resolution and accuracy, making it a reliable source for climate data.

In this study, the historical data was accessed using Google Earth Engine (GEE), a powerful platform for geospatial analysis. GEE facilitated the efficient extraction and processing of the historical rainfall and temperature data, enabling the integration of the historical information with the field data.

### 2.6 Statistical Data Analysis

Statistical tests were conducted to analyze trends in temperature and rainfall in relation to the month of occurrence, peak period and specific periods of observing symptoms from 2020 to 2023. Multinominal logistic regression (MLR) analysis was employed to model the relationship between these climatic factors and the observed categories of the dependent variable which is the specific period of observation. For practical applications, understanding the extremes of spotted wilt intensities—low and high—is crucial for farmers to develop effective management plans and avoid high‐risk situations. Therefore, the expected intensity of spotted wilt for these extremes was analyzed using MLR. The rationale for choosing MLR includes the categorical nature of the dependent variable, which has three distinct categories (no observation, every year, and changes), the ability to handle multiple categories, and the flexibility and robustness of MLR in modeling complex relationships and interactions without assuming linearity. By employing MLR, we aim to gain a deeper understanding of how climatic factors such as precipitation and temperature influence the observed categories of our dependent variable over time, ultimately providing valuable insights for our study and helping farmers make informed decisions to manage tomato wilt virus effectively.

## 3 Findings

### 3.1 Observation of Climate (Rainfall and Temperature Conditions) in the Ada Area From 1960 to 2023

#### 3.1.1 Historical Temperature Pattern

**FIGURE 1:**
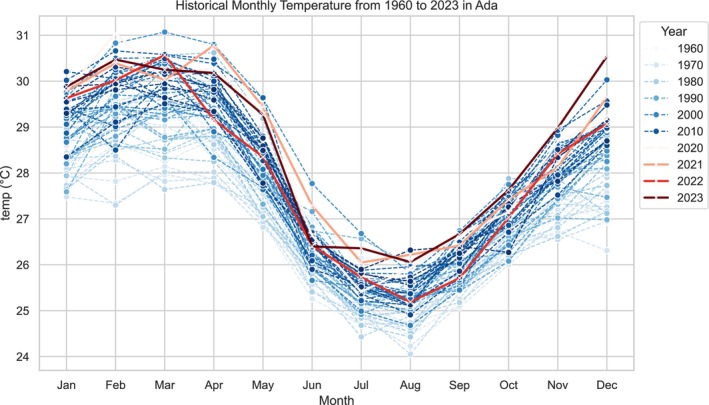
Annual temperatures in Copernicus observations for the periods 1960–2023 for the Ada area.

The historical temperature records for the Ada area, derived from Copernicus data, are presented in Figure 1. The figure shows that temperatures have always exceeded 10°C. Although there were variations in temperature from 1960 to 2023, none of the months during the early spring (i.e., February to March) had temperatures below 10°C. We used 10°C as the threshold for measurement because thrips (eggs), the vector agent that transmit the virus do not develop at temperatures below 10°C (Ganaha‐Kikumura and Kijima 2016; Cao et al. 2018). The Ada area also shows an increasing temperature trend from early autumn to winter (September to December). The result presented in Figure 1 implies that there is no significant change in temperature trend in the Ada area.

#### 3.1.2 Historical Rainfall Pattern

**FIGURE 2:**
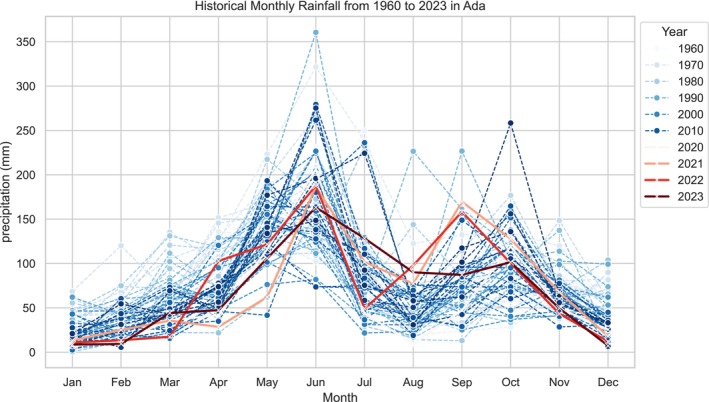
Historical rainfall records in Copernicus observations for the periods 1960–2023 for the Ada area.

The inter‐annual rainfall records for the Ada area are presented in Figure 2. The figure shows high inter‐annual variability, with increasing rainfall peak periods in June. Generally, early spring (i.e., February to March) recorded a low amount of rainfall, a pattern also observed between September and October, followed by a decreasing trend from November to December for the period 1960–2023. Although there are variations in the rainfall pattern for the past 63 years, early spring consistently recorded low rainfall. The Ada area generally experiences approximately 58.19 mm (2.29 in.) of annual rainfall, with rain occurring on about 146.85 days, or 40.23% of the year. Yet, Figure 2 shows that the rainfall pattern has remained relatively stable over the period.

### 3.2 Relationship Between the Occurrence of TSWV and Early Spring Weather Conditions

#### 3.2.1 Records of Spring Temperature in the Ada Area for 2020–2023

Analysis of temperature data from 2020 to 2023 reveals an increase in temperatures from 28°C to 32°C between January and April. Temperature records show a decline from May to September each year. Although 2020 recorded particularly high temperatures from January to April, the overall trend of peak temperatures was consistent across all the years considered in the study. Temperatures tend to rise from January and decrease from June to August over the 4 years under review.

#### 3.2.2 Records of Spring Rainfall in the Ada Area (2020–2023)

**FIGURE 3:**
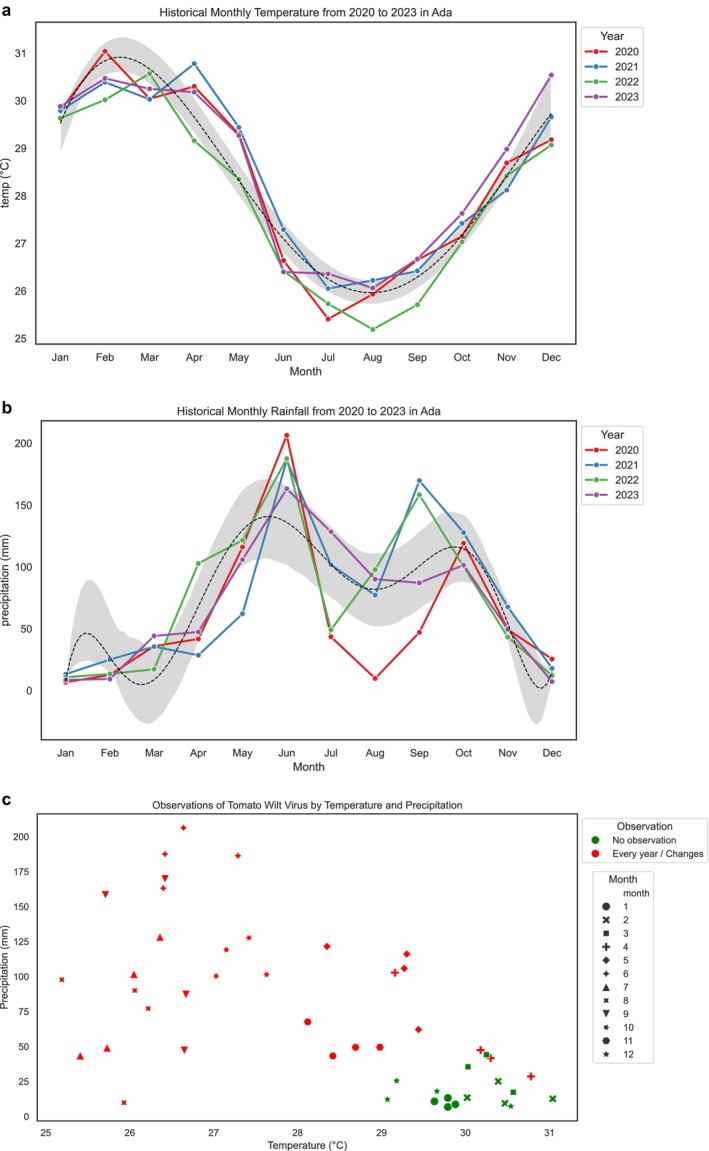
(a) Annual temperatures from 2020 to 2023 in the Ada area. (b) Annual rainfall from 2020 to 2023 in the Ada area. (c) Scatter plot of TSWV observation by temperature and rainfall from 2020 to 2023.

In this study, rainfall below and above 50 mm was selected as the reference point. Over the four‐year period, rainfall was low from January to April, then rose above 50 mm from May to July. It decreased from July to September and increased again from September to November. Historical data (Figure 3a,b) indicate a variance between temperature and rainfall conditions concerning TSWV. Specifically, in the study district, as temperature rises, rainfall decreases, and vice versa. For instance, January recorded less rainfall. The period of interest is from January to April, where we observed low rainfall, while Figure 3b showed a peak period from May to July. A similar trend is observed again for each year during the months September to October for the minor season. The rainfall trend follows a pattern shown by a polynomial curve, with distinct peaks and lows.

### 3.3 Linking Early Spring Temperature and Rainfall Conditions With Farmers' Observations

In this section, we linked findings of data analysis on temperature and rainfall with interviews on farmers' observations on the occurrence of the TSWV as presented in Figure 3c.

Findings from interviews with farmers indicate the period they observe the TWSV each year (2020–2023). In the case of farmers in the Ada area, some farmers planted seeds in the nursery before the first week of March. The period they reported to have observed TSWV was categorized as follows: (1) April; (2) May to June; (3) June to July; (4) July to August; (5) May to September; (6) August to September; and (7) September to October. During interviews, farmers identified symptoms of TSWV using pictures. These symptoms included: leaves developing numerous yellow to brown circular spots on the upper side; parts of leaves appearing bronze with scattered small brown spots; drooping leaves that make the plant look wilted; leaf death; stunted growth; and leaves that roll upwards or downwards, causing the plants to appear stunted or wilted. Farmers who identified the disease from June to August noted that tomato fruits exhibited circular areas, sometimes ringed with small brown spots. Those who observed symptoms from April to June reported that when these symptoms appeared after transplanting, the plants rarely produced fruits. However, farmers acknowledged that some symptoms could also be influenced by the age and nutritional status of the plants.

Farmers' observations of the period of the occurrence of TSWV coincided with the observation of low temperatures and high rainfall records from April to June (i.e., the main farming season) and then from September to November (i.e., minor farming season) in the study district. We plotted farmers' observations about the period of the occurrence of TSWV against temperature and rainfall (Figure 3c) from January to December for 2020 to 2023. We found that in months where temperature records were high, that is 29°C to 31°C, farmers observed TSWV. From our historical data, high temperature records were observed from January to March and from November to December each year. Findings from interviews with farmers indicate that in January to February, they do not observe the symptoms. Starting from March, they sow seeds in the nursery, then they start observing the occurrence of TSWV from the transplanting phase to the maturity phase of the tomato crops in the farm. Their observation also correlates with recorded rainfall occurences.

Findings from farmer interviews on whether TSWV is observed annually revealed two main responses: (1) no observation and (2) occurs every year or changes. In Figure 3c, reports on “no observation” as shown in green for specific months (January to March and November to December) recorded relatively high temperatures (29°C–32°C). We observe that in instances where farmers indicated that they do not observe changes (i.e., no observation of TSWV), the values remained the same. The findings did not show changes between the specific years under consideration (i.e., 2020–2023). This implies that the result is the same whether farmers observe the TSWV twice every year or once a year. Hence, from January to March and November to December, they do not observe the TSWV conditions on their farm when there are high temperatures and low rainfall conditions. The findings in Figure 3c indicate that with low rainfall and high temperature, the possibility of the TSWV occurring and spreading is high. That is why farmers reported that they observed the TSWV starting from May, which confirms our proposition that early spring (i.e., February to March) weather contributes to the occurrence of the TSWV.

Also, we verified from our analysis presented in Figure 3c whether farmers observed the TSWV for the two farming seasons. Their responses indicate that they observed TSWV during the main and minor seasons in the study districts. Farmers who reported “changes” as shown in Figure 3c, did not cultivate tomatoes during the minor season for the specific years under observation. From April to November, farmers observed TSWV across the main and minor seasons. This is because whether it is the main or minor season, the catalyst (i.e., the right conditions) for the virus to thrive is high temperature and low rainfall.

Farmers' accounts also indicate less frequent observation of TSWV on the farm between August and September. The period reported by farmers coincides with records of low temperatures and low rainfall as shown in Figure 3b. Despite findings of low records of rainfall, farmers also reported instances of observing TSWV throughout the year (i.e., main and minor seasons).

#### 3.3.1 Relationship Between Temperature, Rainfall and Month on Observation of TSWV by Farmers

In this study we propose that weather factors, particularly temperature and rainfall, are the important factors that influenced the population of thrips which transmits the TSWV to the tomato crops. The data on the relationship between incidence of TSWV through the abundance of thrips population with weather parameters show a significant relationship, as presented in Table 1. The MLR revealed that both temperature and rainfall are significant contributors. Specifically, higher temperatures decrease the likelihood of both “every year” (*p* < 0.001) and “changes” (*p* = 0.002) categories, while higher rainfall increases the likelihood of both “every year” (*p* = 0.001) and “changes” (p = 0.001) categories. This indicates that temperature and rainfall significantly influence the observations.

**TABLE 1:** Multinominal logistic regression summary.

Variable	Coef (every year)	SE	*z* value	*p* value	Coef (changes)	SE	*z* value	*p* value
Intercept	14.6264	3.456	4.23	< 0.001	15.8288	4.123	3.84	< 0.001
Temperature	−2.1959	−0.567	−3.87	< 0.001	−1.7863	0.578	−3.09	0.002
Rainfall	9.0382	2.789	3.24	0.001	9.1042	2.812	3.24	0.001
Month	−0.3294	0.353	−0.93	0.355	−0.1052	0.312	−0.44	0.732
Year	−0.6465	0.612	−1.05	0.293	−0.6554	0.612	−1.07	0.279

The results are similar for the two categories. Both “every year” and “changes” show the same pattern of significant predictors: temperature and rainfall. The direction of the effects is consistent across both categories, with temperature having a negative impact and rainfall having a positive impact. Since we focused on farmers' observations, specifically the period of occurrence and whether symptoms were observed annually or varied over time, we conducted a multinomial logistic regression using temperature, rainfall, month and year as predictors. The output shows that both temperature and rainfall significantly influence the observations. The finding reinforces the proposition that increasing temperatures and reduced rainfall decreases the likehood of the TSWV of being observed (See Figure 3c).

## 4 Discussion

The average monthly temperatures during early spring (i.e., February to March) and the data generated from farmers indicate a high TSWV occurrence during the period (Figure 3). According to Olatinwo et al. (2008), there is a 100% expected chance of the occurrence of high TSWV when the average daily temperature between February and April is above 28°C. This is because thrips thrive in high temperature conditions between 21°C and 28°C (i.e., warm dry springs). The growth of thrip populations during early warm and dry spring weather conditions (i.e., February–March) and the spread of the TSWV in relation to our findings on high temperature and low rainfall records is relevant for farming because the life‐cycle of the thrip depends on temperature conditions. The virus can only be transmitted if it is acquired from infected plants by larvae thrips (Nachappa et al. 2020). Once a thrip acquires the virus, it circulates and multiplies within the insect, and is transmitted to plants when adult thrips pierce and suck the contents of plant cells (Orecchio et al. 2025). TSWV symptoms may appear on plants within a few weeks after infection (Zhang et al. 2023). Although the window period for thrips to acquire the virus is limited, the wide host range for both the virus and thrips facilitates the development of epidemics (Janssen 2020).

The behavior of the vector thrip and the infection of the TSWV, as indicated in the literature, implies that tomato farms or nurseries are at risk during periods of early spring weather conditions based on farmers' perception and analysis of temperature and rainfall data in 2020–2023. In the study districts, temperatures ranged from 25°C to 31°C throughout the early spring for the periods (2020–2023). As shown in Figure 3c, farmers in the Ada area tend to observe recurring TSWV on their farms when temperatures were between 25°C and 29°C; above that temperature threshold, they reported less observation of TSWV. Studies have also indicated that thrips populations on different crops are positively influenced by temperature and population density increased with increasing temperature. For instance, studies have identified the correlation between high temperatures and the occurrence of thrips and TSWV in peanut fields (Olatinwo et al. 2008, 2009; Nuti et al. 2014; LaTora et al. 2022); onion fields (Waiganjo et al. 2008; Karuppaiah et al. 2023); Tabacco (Morsello et al. 2008; Mila 2011; Chappell et al. 2013); watermelon crops (Barbosa et al. 2019); mango orchards (Pearsall and Myers 2001; Aliakbarpour et al. 2010); and strawberry crops (Sampson et al. 2021). Beside these crops, a study conducted by Riley et al. (2012) also showed the temporal relationship of thrips populations to TSWV incidence in tomato fields, and the findings show high temperatures influence the growth of thrips. The evidence identified in other studies corroborate our findings on the correlation between the occurrence of TSWV and the weather parameters (i.e., rainfall and temperature) analyzed in this study.

The analysis from Figure 3c indicates that the high temperature (25°C–32°C) recorded between January–April correlates with farmers' observation (Figure 3c). Infestation of the virus by thrips could have been initiated from March and continued up to July. The inferences that could be made from the findings of the study are that thrip presence and weather parameters (i.e., rainfall and temperature) have a strong significant positive relationship between farmers' observation on the occurrence of the symptoms of thrips infestation and maximum temperature. This is because interviews with farmers indicated that they observe symptoms (see Section 3.3) from the month of March to July (i.e., main farming season) and October—November (i.e., minor farming season). However, between August and September these symptoms were not observed. Their observation corroborates the temperature conditions recorded in the study area. With regards to this finding, Waiganjo et al. (2008) also identified that dry weather (30.3 mm rainfall) with moderately high temperatures (15.6°C–28.2°C) increased seasonal thrips numbers leading to the records of TSWV conditions among onion crops. Temperatures above 35°C and drought have been reported to be unfavorable to the survival of thrips, resulting in population decline (Varadharajan and Veeraval 1995). In our study, temperatures ranged from 25°C to 32°C throughout the 4 years period, implying a favorable condition for the survival of thrips and the transmission of the virus to the tomato plants.

A significant negative correlation was found with the occurrence of TSWV and rainfall. The results corroborate findings of Ibrahim and Adesiyun (2010) who carried out a study on the effect of rainfall on the control of onion thrips, which found that heavy rainfall has a negative effect on the population of thrips, either through suppression of adult flight or by killing thrips larvae. A finding by Morsello et al. (2010) also shows that the influence of precipitation on the spring dispersal of *Frankliniella fusca* changes as the season progresses. Heavy rainfall can suppress thrips populations by increasing the mortality rate, thereby reducing the population growth rate, or by suppressing the flight of adults (Norris et al. 2002). This finding was also identified by Kirk's (2004) study on the linkages between cereal thrips and thunderstorms. It is also possible that the positive effect of frequent rain on thrips populations might offset the occurrence of the TSWV. However, tomato plant interactions with other agronomic factors or weather variables, such as evapotranspiration and temperature, could influence the impact of the thrips population. Hence, although our findings show that low rainfall records in early spring (i.e., February to March) could result in high incidence of TSWV due to thrips population, their intensity, frequency, and amount play relevant roles in the spread of the virus.

We also found that the chances of TSWV infesting seedlings in the nursery in March or April before transplanting to the farm were high, as thrip movement is predominant during high temperatures and their egg take 2–4 days to survive under high temperature before developing into first instar larva (Sherwood et al. 2003). Therefore, a daily temperature above 28°C during February and March could increase the intensity of TSWV when seeds are planted in the nursery from February or March, particularly by irrigation farmers. Thrips may become active or reproduce at this temperature. We observed that the periods where high temperatures were recorded from 2020 to 2023 coincided with farmers' observations about the occurrences of the symptoms of the virus. This implies that the crops might have been infested with the virus through the vector thrips during periods the seedlings were in the nursery. Generally, temperatures above 21°C were recorded from January–April for the period 2020–2023. The findings of the study could imply that in extreme high temperature in regions such as the Ada area, thrip population could even be high throughout the year. Based on these findings, we confirm the proposition that the TSWV starts developing from March or April from the nursery before transplanting to the farm.

For virus‐related diseases, it takes time for the symptoms to manifest for farmers to spot in the field. This informs findings that farmers' observation on the occurrence of the TSWV conditions in the farm between April and July, with the month of severity occurring in June. The findings imply that the virus might have been transmitted in the crops during early spring (February–March) when the seedlings were in the nursery. Therefore, the period where farmers observe the symptoms in the field was mostly during the flowering or fruiting stage. Flowering of tomatoes occurs mostly under moderate daily temperatures (Sato et al. 2001). Farmers' assertion of the timing of the visible symptoms corresponds with Lemtur and Choudhary's (2016) study, which also reported that “The peak of thrip population was reached at flowering time of the crop on all the studied genotypes”. The findings of the study imply that while the crops may be under the infestation of TSWV, their fruit formation could also be affected by high temperatures.

The growth of flowers on the crop could also provide a conducive environment for perpetuating thrips through quality feeding and breeding place (Moanaro and Choudhary 2016), especially during high temperatures and periods of dry spells in between the season (July–August). Hence, although there is a probability that TSWV could start in the nursery, there are chances that a continual increase in temperature in the main season (i.e., summer/May–August) and the absence of rainfall for some days during the period could also result in thrip population build‐up which could lead to the spread of the virus in crops that have been transplanted to the field.

Although the focus of the study is on the effects of early spring weather conditions on the risk of TSWV occurrence through increase in thrip population, we also observed that there is a high risk of TSWV infestations in the minor season (i.e., early autumn to winter). This is because there is an identified weak increasing trend in rainfall occurrence with peak periods mostly occurring in October. The TSWV conditions experienced in early autumn to winter (September–November) could also explain why some farmers reported during interviews to cultivate tomatoes for only one season to avoid the vagaries of the weather.

Generally, high temperatures are not conducive to the growth of tomatoes, particularly in conditions where smallholders operate without a greenhouse to protect the crops from the vagaries of the weather. Research findings from other studies in Ghana show that the growth of tomatoes under high temperatures and humidity is an environmental factor that has retarded the cultivation of tomatoes in Ghana (Guodaar et al. 2017). Asare‐Bediako et al. (2007) identified that high humidity and abundant rainfall in Ghana create favorable conditions for pests and diseases, acting as impediments to successful tomato cultivation. Lamptey et al. (2013) also investigated viral diseases prevalence in major tomato‐growing areas and identified the role of varied high and low weather conditions that play roles in the occurrence of pathogens. Although we analyzed 63 years of data on temperature in the Ada area (see Section 3.1), the increasing trend suggests that in the future, the occurrence of extreme temperatures may have implications on tomato yield, which is sensitive to temperature increase and the incidence of thrips. The findings of the study show that high temperatures constrain tomato production in the district, leading to low yield, loss of production, high cost of production, and its impacts on food availability and nutrition security.

## 5 Conclusion

The objective of this study is to examine the occurrence of early spring (i.e., February to March) weather conditions and its effects on the risk of TSWV occurrence. The TSWV is a biotic factor that severely affects tomato production globally because it occurs across temperate, tropical, and subtropical regions, impacting a wide variety of crops and causing substantial economic losses worldwide. Our findings from the multinomial logistic regression analysis show a high correlation between temperature and the occurrence of TSWV. It indicates that high temperature (29°C—32°C) recorded between January to April correlates with farmers' observation on the occurrence of TSWV. The inferences that could be made from the findings of the study are that TSWV and weather parameters (i.e., rainfall and temperature) have a strong significant positive relationship. The findings that the observation of TSWV by farmers started from March—July could also imply that the virus could have occurred in the nursery and have been transported to the farm, leading to the continual transmission of the virus among the crops throughout the farming seasons. The favorable rainfall and temperature conditions may also enhance thrip vector populations in the farm, leading to the persistent incidence of TSWV in the study district. The key conclusion drawn from the findings is that weather trends, that is, rainfall and temperature can be used to determine the potential occurrence of TSWV and its management in tomato production.

### 5.1 Implications of the Findings for Practical Application by Farmers, Extension Officers and Policy

The results of the study suggest that climatic trends can be used by agricultural extension officers and plant health experts to determine thrip control strategies such as nursery treatments and prevention of virus transmission by thrips in the nursery before transplanting. Farmers can be encouraged to use natural farming methods such as crop rotation with non‐host crops, setting sticky traps, removal of infected crops, weeding along headlands and irrigation channels, application of botanical extracts as insecticides, planting healthy seeds and resistant varieties, and prompt removal and destruction of old tomato plants and other host crops after harvest.

Furthermore, treatment of the conditions should start from the nursery. Particularly, farmers should be trained on how to scout for the presence of thrips when the seedlings are in the nursery to help instigate management measures at early stages before the spread of the virus to other crops in the field.

Agricultural extension officers should co‐develop a method of risk assessment of TSWV to facilitate integrated management with farmers to enhance adoption of management practices.

The findings of the study suggest the need to provide timely daily and monthly weather forecast information services to enable farmers to carry out management practices that could prevent the infestation of the TSWV in the study area and other similar contexts. These roles could be fulfilled by stakeholders in the national meteorological and agricultural sector.

### 5.2 Gaps for Future Research Analysis

The findings of the research also leave gaps for future research on the sector. The study relied on accumulated weather data for the entire district without using weather records from specific farms. Although the virus is thrip‐transmitted, we did not monitor thrips population dynamics directly on the farm. We propose that future research consider monitoring thrip populations.

Future studies could also collect georeferenced points, rainfall and temperature data from specific farms to conduct spatial and temporal analysis concerning the movement of thrips, which are host to the TSWV and other multivariate factors that condition the spread of pathogens in tomato fields.

In our study, farmers' symptom identification relied on visual confirmation with pictures. Since symptoms like leaf spots and wilting may also arise from other biotic or abiotic stresses, we proposed that future research analyses should validate TSWV presence through confirmatory diagnostics (e.g., molecular assays or serological tests). Implementing such validation would considerably enhance the robustness of the association between climatic variables and viral incidence.

The study could not gather meteorological data from specific farms (i.e., based on field locations and the nearest weather station) that monitors several weather parameters, including air temperature, precipitation, relative humidity, solar radiation, wind speed and direction, soil moisture, and soil temperature. The study could not also analyze varieties of rainfall conditions such as number of rainy days, intensity of rainfall, and amount of rainfall in a month. Future study could focus on other weather risk indexes including varied rainfall conditions to ascertain the TSWV occurrence. Although we derived knowledge on the severity of the occurrence of TSWV, this was based on farmers’ perception (experienced based recall). A future study could develop severity risk indicators such as crop loss or number of crops infected based on acreage in a season to assess TSWV severity, such as: “not severe”; “moderate severe”; “highly severe”; and “very highly severe.” Future research could also focus on measuring TSWV severity of occurrence through using insect traps to identify vector thrip population growth in relation to temperature and rainfall occurrence during the farming season. A future study could focus on co‐produce TSWV risk indexes with different typologies of farmers to support weather‐based pest forecasting for effective crop protection. The analysis could provide practical findings on how weather information can enable farmers to make proactive decisions to mitigate the spread of TSWV in the area.

Also, a study that applies Artificial Intelligence using an Agent‐Based model to analyze farmers' decisions using management interventions. A real‐life research initiative could also introduce the use of resistant plants carrying the Sw5 gene to determine its performance under varied weather conditions in the study district or other contexts. The results from these future initiatives could be helpful to inform policymakers and agricultural extension agents in managing the TSWV and thrips. The findings can also be disseminated to farmers and stakeholders through training workshops.

## Funding

This research was conducted under the sponsorship of the Alexander von Humboldt fellowship and Bayer Foundation Fellowship program. However, the views expressed herein are the sole responsibility of the authors.

## Conflicts of Interest

The authors declare no conflicts of interest.

## Supporting information


**Appendix S1:** Supporting Information.

**Data S1:** Supporting Information.

## Data Availability

All the data used for the study have been included in the manuscript.
