# Supplementary material for: Weather Conditions and the Risk of Tomato Spotted Wilt Virus (TSWV) in Tomato Producing Areas in Southern Ghana
Source: Plant Environ Interact. 2026 Feb 16;7(1):e70121. doi: 10.1002/pei3.70121 (PMC12909278; doi:10.1002/pei3.70121)
Supplement: Supplementary file 1 — Appendix S1: Supporting Information. [file PEI3-7-e70121-s002.docx]

**Appendix 1: Supplementary Material**

**QUESTIONAIRE**

- 1. Household’s consent obtained [________] 1=YES 0=NO
  2. If No (1.1),why?___________________________________ (End Survey)

**SECTION A:FARMER INFORMATION.**

**1.0 Household Information.**

| A1 | Questionnaire ID |  |
| --- | --- | --- |
| A2 | Date of the interview (dd.mm.yy) |  |
| A3 | Start time |  |
| A4 | Enumerator name |  |
| A5 | Respondent Name (three names): |  |
| A6 | Gender of the household head | 1=Male  0=female) |
| A7 | Respondent Name (three names): |  |
| A8 | Age of the Respondent | Years |
| A9 | Name of household head: |  |
| A10 | Sex of household head | 1=Male  0=Female |
| A11 | Size of the Household |  |
| A12 | Highest Level of Education | Years Spend in School |
| A13 | Phone number (of household head) |  |
| A14 | District |  |
| A15 | District zone |  |
| A16 | Village |  |
| A17 | Type of farming | Rainfed  Irrigation  Both Rainfed and Irrigation |

**Land ownership**

1. **How many plots does the household have, including gardens?**
2. **P1**
3. **P2**
4. **P3**
5. **P4**
6. **P5**
7. **P6**

**For the plot which you consider to be your main plot, ………..**

1. What is the tenure arrangement of the main plot?
2. **Owned and worked by household members ◊ Skip to the next section**
3. **Owned and sharecropped out ◊ go to 5**
4. **Not owned, rented in ◊ go to question 5**
5. **Not owned, sharecropped ◊ go to question 5**
6. **Not owned, borrowed ◊ Skip to the next section**
7. If you rent or/and sharecrop, what is the payment arrangement?
8. **Seasonal**
9. **Annual**
10. **Others (Specify) ……**
11. How much area was cultivated with this crop under discussion? **[Verify that this is not greater than the total plot size in question above] UNITS OF MEASURE: 1-Hectare 2-Square meter 3-Acre**

**Area: ……Unit: ……**

**Cost of seed**

1. **What type of seeds do you primarily use for cultivation? (Record the name of the variety)**

**.................................. (LIST OF VARIETIES AVAILBLE IN KISUMU AND THEIR COST)**

6. How do you usually acquire your seeds?

**A. Purchase from agro-dealers**

**B. Government or NGO distribution**

**C. Saved from previous harvest**

**D. Cooperative or group purchase**

**E. Other (please specify)**

1. What is your average cost of seed per the size of the land we are discussing about?

**..............................................**

8. How often do you purchase new seeds?

**A. Every planting season**

**B. Once a year**

**C. Every 2–3 years**

**D. Rarely, mostly reuse seeds**

**E. Not applicable**

1. How many times do you plant tomatoes per farmland in a year?
2. 1
3. 2
4. 3
5. Do you recognise the disease condition affecting the tomato plants in any of these pictures? Yes No


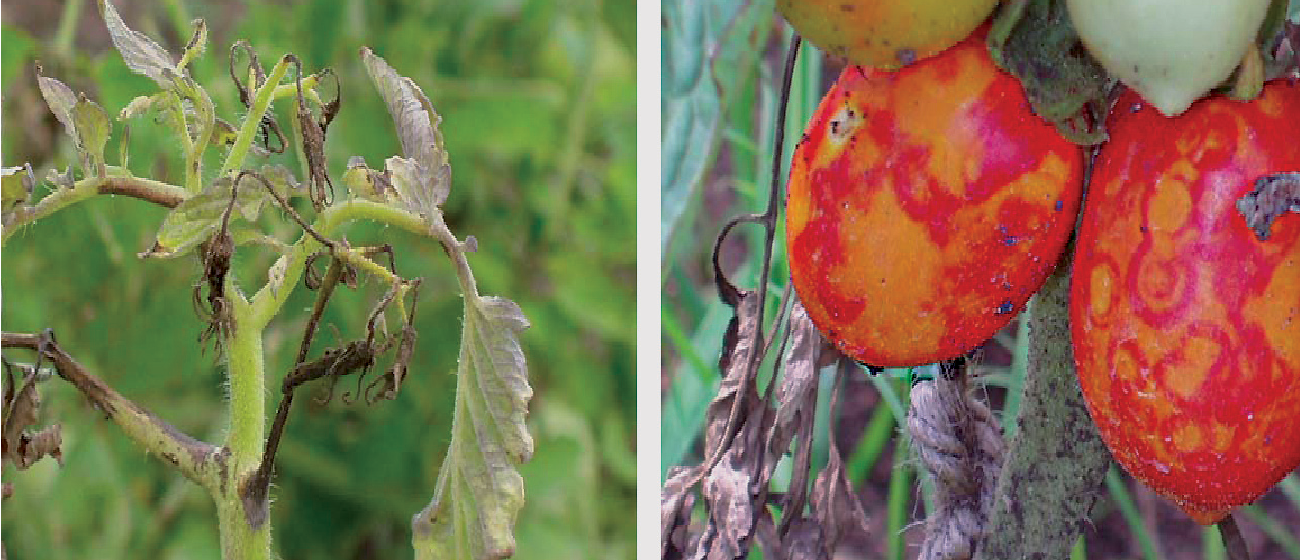


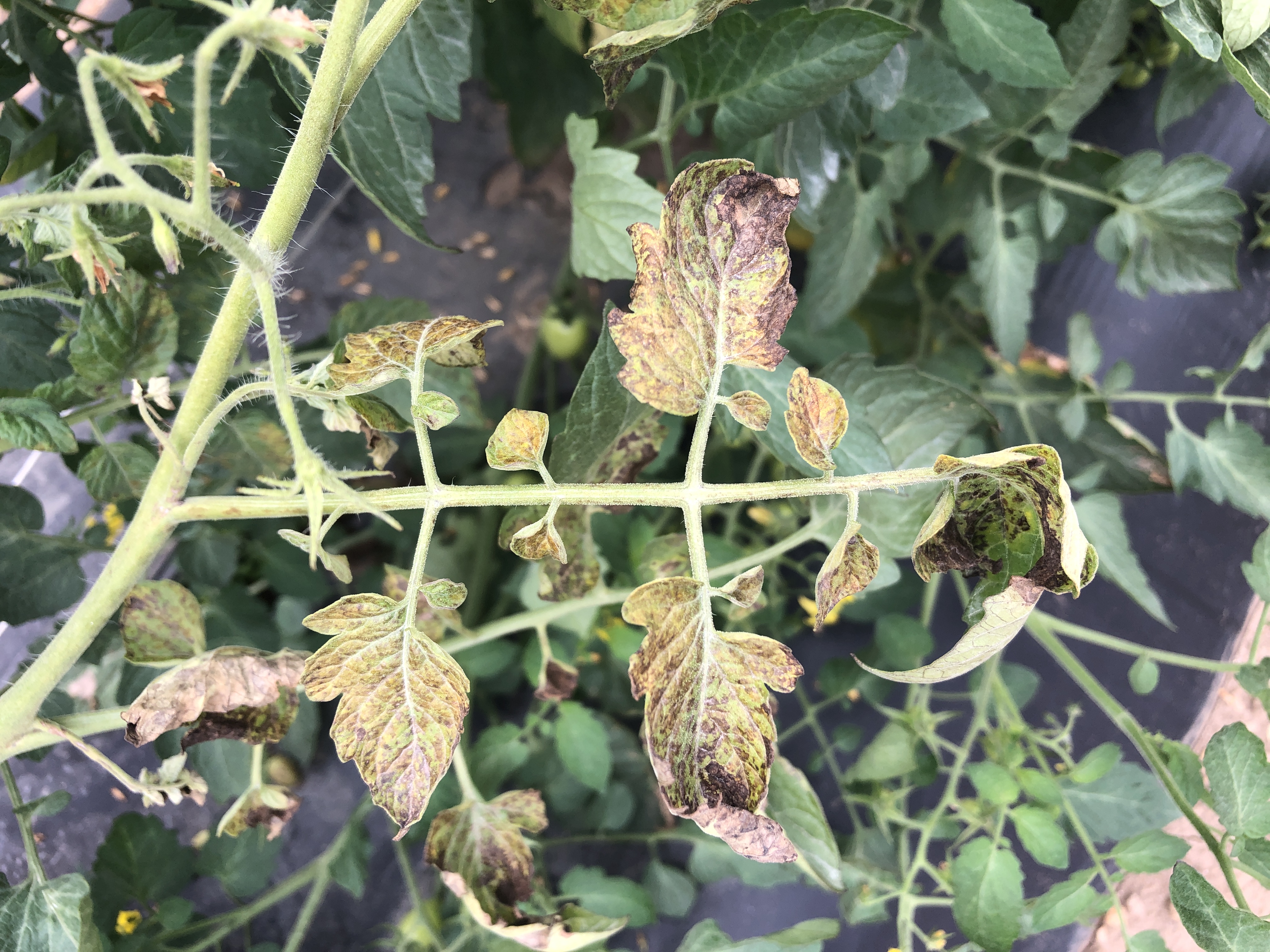


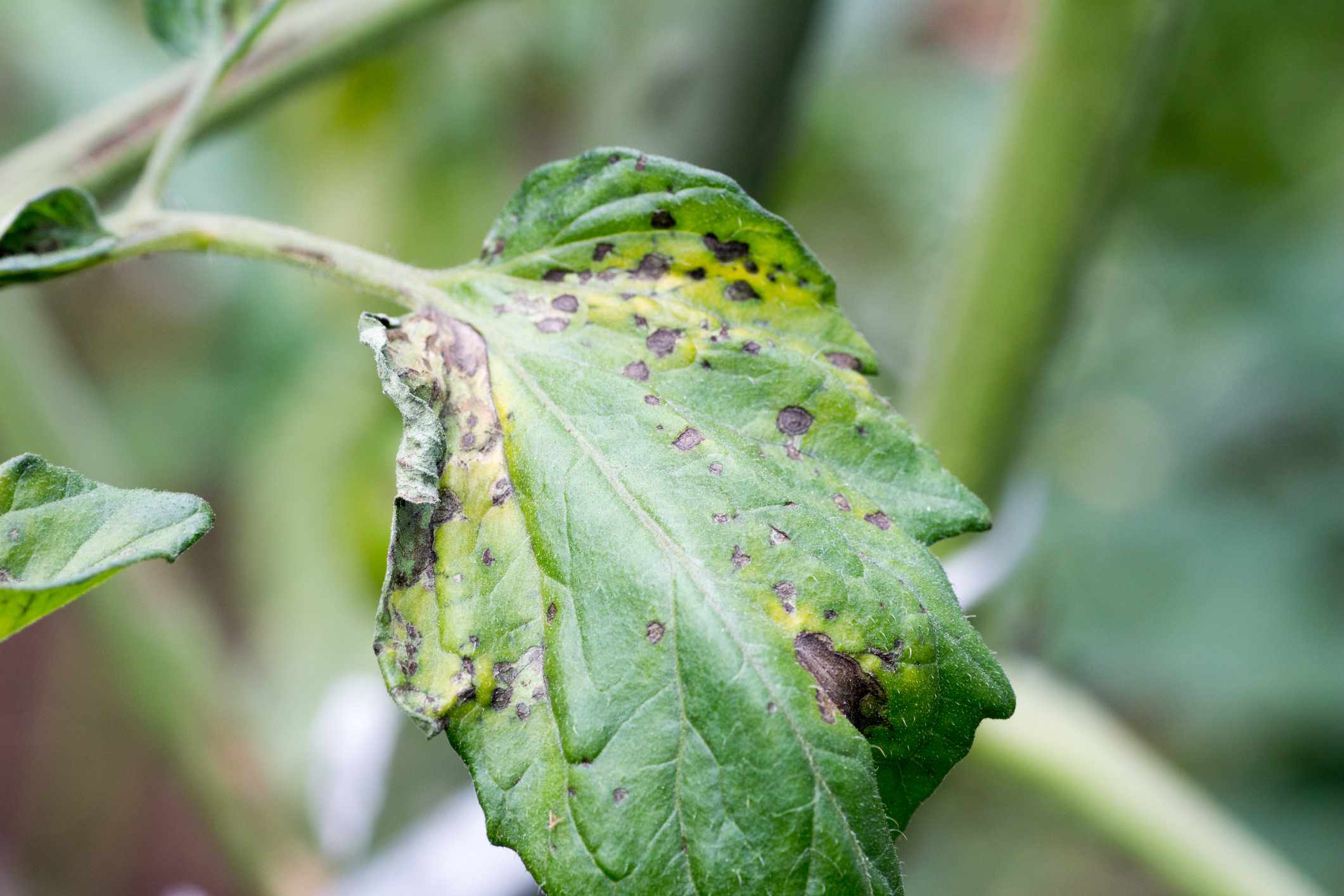


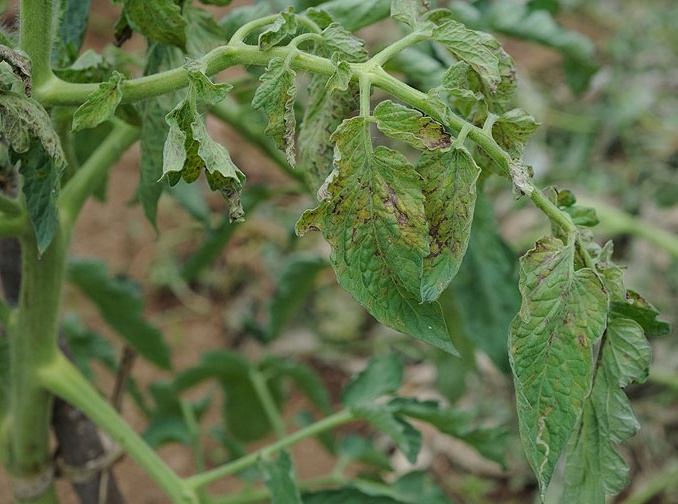


If yes, continue with the following questions

1. What time (month) does it occur within the year?
2. What time do you **observe the peak** of the symptoms of the disease (referring to the changes in the colours of the leaves, and the fruits)?
3. In your opinion, what do you think are the causes of the conditions in the picture?
4. Do you observe the symptoms each time you plant or is there any specific season that you spot it?
5. How long have you made the observation of the disease on your farm? (Tick or circle)

| **Year** | **Season 1 (Main season)** | **Season (Minor season)** |
| --- | --- | --- |
| 2020 | Yes/No | Yes/No |
| 2021 | Yes/No | Yes/No |
| 2022 | Yes/No | Yes/No |
| 2023 | Yes/No | Yes/No |

1. Can you grade the severity of the disease
2. **Low**
3. **Mild**
4. **High**
5. **Very high**

Thank you for responding to the questions
